# Establishment of patient derived xenografts as functional testing of lung cancer aggressiveness

**DOI:** 10.1038/s41598-017-06912-7

**Published:** 2017-07-27

**Authors:** Massimo Moro, Giulia Bertolini, Roberto Caserini, Cristina Borzi, Mattia Boeri, Alessandra Fabbri, Giorgia Leone, Patrizia Gasparini, Carlotta Galeone, Giuseppe Pelosi, Luca Roz, Gabriella Sozzi, Ugo Pastorino

**Affiliations:** 1 Tumor Genomics Unit, Department of Experimental Oncology and Molecular Medicine, Fondazione IRCCS Istituto Nazionale dei Tumori, Milan, Italy; 2 Department of Pathology and Laboratory Medicine, Fondazione IRCCS Istituto Nazionale dei Tumori, Milan, Italy; 3 Thoracic Surgery Unit, Department of Surgery, Fondazione IRCCS Istituto Nazionale dei Tumori, Milan, Italy; 4 Department of Clinical Sciences and Community Health, Università degli Studi di Milano, Milan, Italy

## Abstract

Despite many years of research efforts, lung cancer still remains the leading cause of cancer deaths worldwide. Objective of this study was to set up a platform of non-small cell lung cancer patient derived xenografts (PDXs) faithfully representing primary tumour characteristics and offering a unique tool for studying effectiveness of therapies at a preclinical level. We established 38 PDXs with a successful take rate of 39.2%. All models closely mirrored parental tumour characteristics although a selective pressure for solid patterns, vimentin expression and EMT was observed in several models. An increased grafting rate for tumours derived from patients with worse outcome (p = 0.006), higher stage (p = 0.038) and higher CD133^+^/CXCR4^+^/EpCAM^−^ stem cell content (p = 0.019) was observed whereas a trend towards an association with SUV_max_ higher than 8 (p = 0.084) was detected. Kaplan Meier analyses showed a significantly worse (p = 0.0008) overall survival at 5 years in patients with grafted vs not grafted PDXs also after adjusting for tumour stage. Moreover, for 63.2% models, grafting was reached before clinical recurrence occurred. Our findings strengthen the relevance of PDXs as useful preclinical models closely reflecting parental patients tumours and highlight PDXs establishment as a functional testing of lung cancer aggressiveness and personalized therapies.

## Introduction

Preclinical models are a unique component in almost every aspect of translational cancer research. They are suitable and deeply exploited for understanding the biological complexity of the disease, but also for developing new treatments. Conventional cell lines are widely used as preclinical models, both *in vitro* and *in vivo* as xenografts (XGs) and, currently, the NCI-60 cancer cell line panel is the most widely used and best characterized collection of human cancer cells for preclinical drug screening experiments^1^. However, cell lines represent an unsatisfactory preclinical model for drug testing due to the lack of predictive value in specific cancer types. This is probably due to a progressive drift of genetic alterations and cellular subpopulations driven by culturing processes^2–4^. Furthermore, XGs grow subcutaneously in mice as a compact mass of cancer cells, without resemblance of the original cellular pattern and stromal structure^5^. Patient derived xenografts (PDXs) models represent a “never *in vitro*” model that maintains a more faithful tumour-stroma architecture avoiding the bias of culture-driven genetic drift. PDXs have been reported for many different types of solid tumours^6–12^ and have demonstrated their utility in predicting efficacy of a range of therapeutic approaches (reviewed in ref.^13^). Among them, colon cancer PDXs led to very interesting preclinical discoveries, such as the identification of HER2 as an effective therapeutic target in cetuximab-resistant colorectal cancer^14,15^. Lung cancer is the leading cause of cancer deaths worldwide accounting for 1.6 million new cases annually and, because of its poor prognosis, for 1.38 million deaths each year (18,2% of all cancer deaths^16^). In particular, non-small cell lung cancer (NSCLC) is poorly chemosensitive and the use of target therapies is limited to about 15% of patients where a specific target lesion is observed^17^. Therefore, the identification of additional target subpopulations is essential and PDXs represent a unique preclinical model for this purpose. Establishment of lung cancer PDXs (LcPDXs) has already been reported by other groups who mainly pointed to the analysis of grafting determinants and maintenance of primary tumour’s features in early passages in mice^18–24^.

Here, we report the establishment of a large panel of LcPDXs derived from NSCLC patients, which closely recapitulate and maintain primary tumour features over 10 passages in mouse. Interestingly, we found significant correlations between PDX take rate and patient’s prognosis and survival suggesting PDX grafting as a surrogate functional testing to anticipate lung cancer aggressiveness. Targeted mutation sequencing showed maintenance of genetic profile in LcPDX compared to respective parental tumours. Finally, the analysis of growth characteristics allowed us to identify among LcPDXs a group of faster growing models that become stable in mice before disease recurrence in patients. These group of LcPDX may be utilized as “avatars” for testing personalized treatment strategies.

## Results

### Establishment of a lung cancer PDX perpetual bank

Tumour samples from 97 lung cancer patients (65 adenocarcinomas - ADC, 16 squamous cell carcinomas - SCC and 16 other lung tumours or metastases - OL) have been implanted in both flanks of SCID or nude mice. Overall, 49/97 (50.5%) samples successfully gave rise to a P1 generation in mice. However, grafted PDXs showed variable and generally slower growth rate during the first three passages in mice (P1–P3) and 11 of them were lost in this time frame. In particular, latency period and time to transplantation were generally higher and more heterogeneous in P1–P3 period, whereas beyond P3, PDX growth was more homogeneous (Supplementary Figure 1). At P3, 38 PDXs (39.2%) were considered grafted and suitable for further analysis. In detail, 23/66 ADC (34.8%); 7/16 SCC (43.8%) and 8/15 OL (53.3%), including 2 large cell, 1 sarcomatoid, 3 small cell carcinomas, 2 lung metastases from sarcoma and oral cavity carcinoma successfully grafted (Tables 1 and 2). Furthermore, we set up a freeze/thawing procedure on tumour samples derived from established PDXs that was thoroughly exploited to establish a large and continuously growing collection of frozen samples (Supplementary Table 1).

**Table 1:** Patients and tumours characteristics, overall and according to graft.

	All subjects (n = 97)	Graft		
		Yes (n = 38)	No (n = 59)	p-value*
***Patients characteristics***				
Sex				
Female	35 (36.1)	16 (45.7)	19 (54.3)	*0*.*322*^1^
Male	62 (63.9)	22 (35.5)	40 (64.5)	
Age				
*Mean (sd) – Median (IQR)*	65.3 (9.1)–67.0 (60.0–72.0)	63.3 (9.9)–65.0 (58.0–71.0)	66.6 (8.3)–69.0 (61.0–73.0)	*0*.*137*^*2*^
Smoking habits				
Never	9 (9.3)	4 (44.4)	5 (55.6)	*0*.*349*^*1*^
Ex	46 (47.4)	21 (45.7)	25 (54.3)	
Current	42 (43.3)	13 (30.9)	29 (69.1)	
Pack years				
≤	46 (47.4)	22 (47.8)	24 (52.2)	*0*.*098*^1^
40 pack-years				
>40 pack-years	51 (52.6)	16 (31.4)	35 (68.6)	
*Mean (sd) – Median (IQR)*	46.8 (31.4)–44.0 (30.0–60.0)	44.4 (36.6)–40.0 (23.3–55.0)	48.4 (27.7)–50.0 (30.0–70.0)	*0*.*148*^*2*^
FEV1**				
*Mean (sd) – Median (IQR)*	89.5 (20.4)–90 (78.5–102.5)	91.8 (18.2)–92.5 (84.0–104.0)	88.0 (21.6)–89.0 (77.0–101.5)	*0*.*323*^*2*^
FEV1/FVC**				
*Mean (sd) – Median (IQR)*	71.7 (10.1)–71.0 (65.0–78.5)	72.6 (10.3)–74.0 (66.0–80.0)	70.2 (10.0)–71.0 (64.0–77.0)	*0*.*278*^*3*^
COPD				
No	39 (42.9)	16 (41.0)	23 (59.9)	*0*.*805*^1^
Yes	52 (57.1)	20 (38.5)	32 (61.5)	
Missing	6			
***Outcome***				
Mortality				
Alive	55 (56.7)	15 (27.3)	40 (72.7)	*0*.*006*^*1*^
Dead	42 (43.4)	23 (54.8)	19 (45.2)	
Disease				
No	45 (46.9)	11 (24.4)	34 (75.6)	*0*.*008*^*1*^
Yes	51 (53.1)	26 (51.0)	25 (49.0)	
Missing	1			
***Tumour characteristics***				
SUV				
≤5	21 (23.9)	7 (33.3)	14 (66.7)	*0*.*418*^1^
>5	67 (76.1)	29 (43.3)	38 (56.7)	
≤8	39 (44.3)	12 (30.8)	27 (69.2)	*0*.*084*^1^
>8	49 (55.7)	24 (49.0)	25 (51.0)	
Missing	9			
*Mean (sd) – Median (IQR)*	10.2 (7.0)–8.7 (5.4–13.8)	11.0 (6.2)–11.9 (6.5–15.1)	9.6 (7.4)–7.6 (4.9–12.9)	*0*.*109*^*2*^
Stage				
I	35 (36.1)	9 (25.7)	26 (74.3)	
II	21 (21.6)	8 (38.1)	13 (61.9)	*0*.*076*^*1*^
III/IV	41 (42.3)	21 (51.2)	20 (48.8)	
TNM Staging**				
N = 0	46 (50.0)	14 (41.2)	32 (55.2)	
N > 0	46 (50.0)	20 (58.8)	26 (44.8)	*0*.*279*^*1*^
Subtype				
Adenocarcinoma	66 (68.0)	23 (34.9)	43 (65.2)	*0*.*383*^*1*^
Squamous cell carcinoma	16 (16.5)	7 (43.8)	9 (56.2)	
Other	15 (14.5)	8 (53.3)	7 (46.7)	
***CD133*** ^**+**^ ***cells*** ^***4***^				
>=1%	31 (54.4)	18 (56.3)	13 (52.0)	*0*.*7492*^*1*^
<1%	26 (45.6)	14 (43.7)	12 (48.0)	
Missing	40			
***CD133*** ^**+**^ ***/CXCR4*** ^**+**^ ***/EpCAM*** ^***−***^ ***cells*** ^***4***^				
Yes	28 (58.3)	21 (77.8)	7 (33.3)	*0*.*0019*^*1*^
No	20 (41.7)	6 (22.2)	14 (66.7)	
Missing	49			

*P for group comparison: ^1^Chi-square test; ^2^Wilcoxon’s rank-sum test; ^3^Student’s t-test; ^4^PDXs values; **5 missing values. SUV: Standard Upatake Value; COPD: Chronic Obstructive Pulmonary Disease.

**Table 2:** Hazard ratios (HR) and 95% confidence intervals of overall survival and disease free survival according to tumour graft, age, sex, stage and SUV.

	Overall survival		Disease free survival	
	crude HR (95% CI)	adjusted** HR (95% CI)	crude HR (95% CI)	adjusted** HR (95% CI)
Engrafting PDXs (yes vs no)	2.73 (1.48–5.04)	2.61 (1.37–4.98)	2.41 (1.38–4.22)	1.90 (1.04–3.48)
Sex (men vs women)	1.13 (0.60–2.15)	1.07 (0.52–2.23)	1.05 (0.59–0.88)	0.95 (0.49–1.85)
Age (1 year increment)	1.02 (0.99–1.06)	1.03 (0.99–1.07)	1.02 (0.99–1.05)	1.01 (0.98–1.05)
Stage				
II vs I	2.79 (0.89–8.80)	2.65 (0.79–8.89)	4.24 (1.59–11.30)	5.13 (1.83–14.41)
III/IV vs I	8.93 (3.43–23.27)	8.59 (3.13–23.60)	9.09 (3.74–22.07)	8.84 (3.48–22.50)
Subtype				
Squamous cell vs adenocarcinoma	0.78 (0.30–2.04)	0.85 (0.29–2.49)	1.31 (0.62–2.75)	1.67 (0.69–4.02)
Other vs adenocarcinoma	1.75 (0.82–3.71)	1.41 (0.62–3.19)	1.70 (0.83–3.46)	1.49 (0.70–3.21)
SUV (>8 vs <=8)	1.81 (0.96–3.40)	1.25 (0.62–2.52)	2.28 (1.25–4.14)	1.51 (0.78–2.91)

**Estimated from Cox proportional hazard regression model adjusted for engrafting PDXs, sex, age, tumour stage, tumour subtype, and SUV.

### LcPDX models recapitulate primary tumour features

#### Histopathology

**Figure 1:**
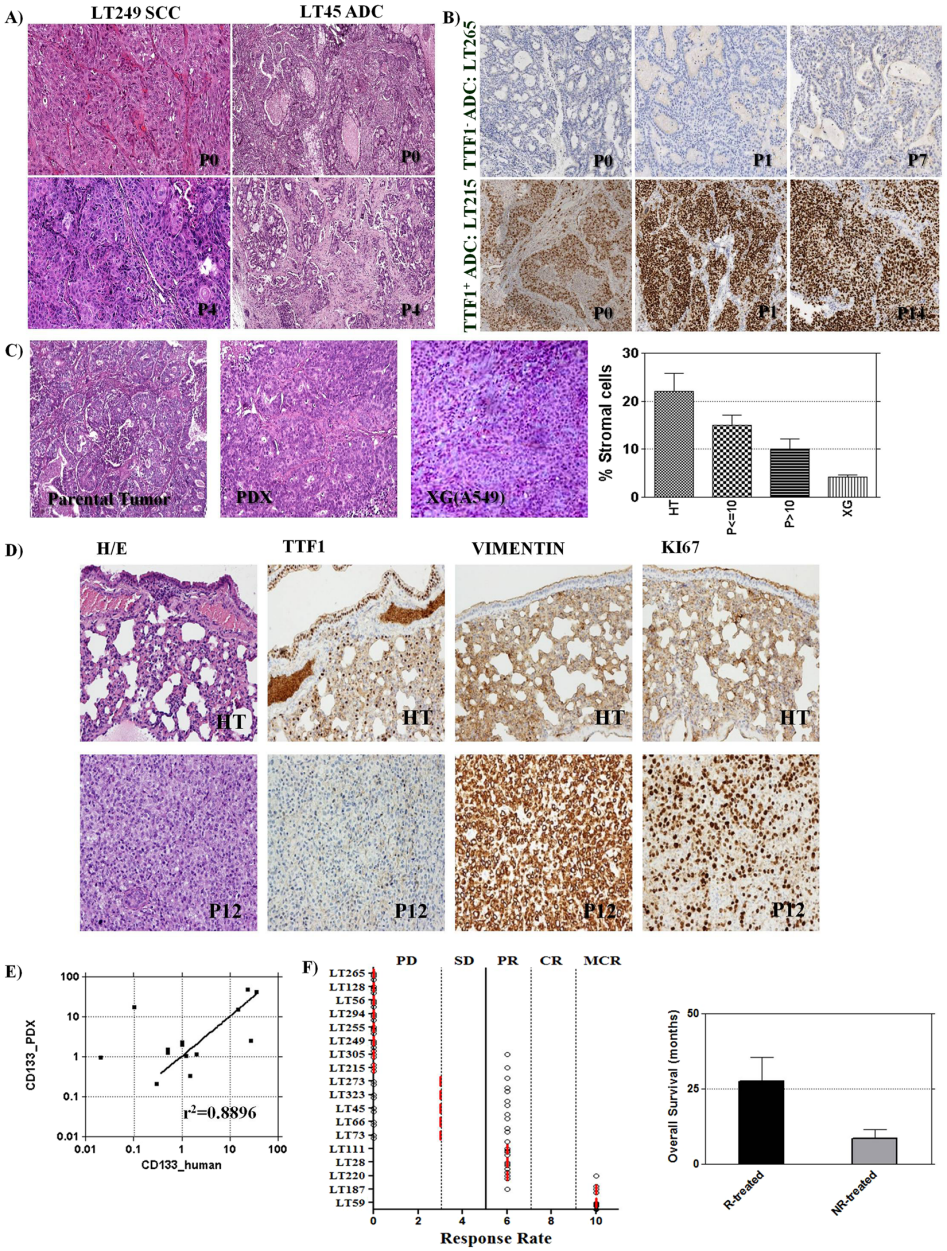
Analysis of PDXs characteristics. (**A**) Parental tumour diagnosis was maintained in all established PDX. Pictures show a representative example of 1 ACC- and 1 SCC-derived PDXs. (**B**) Immunohistochemistry markers analysis confirmed that our PDXs platform generally maintained the same expression pattern of parental tumours, picture shows a representative example of a TTF1 expressing ADC-derived PDX. (**C**) PDXs stromal content was generally lower compared to parental tumours, especially at P > 10. However, stromal components were always more represented in PDXs than in cell line derived xenografts (average stromal component: 22 ± 12% in parental tumours, 15 ± 7% in P ≤ 10 and 10 ± 7% in P > 10, n = 10; 4.2 ± 1.012% in lung cancer cell line xenografts, n = 5). (**D**) In PDX LT220 complete loss of TTF1 and marked acquisition of vimentin and Ki67 labelling index was observed along with the appearance of spindle cells. ADC = Adenocarcinoma, SCC = Squamous Cell; (**E**) CD133^+^ cell percentage was also maintained in PDX models (n = 17, p = 0.0005, r^2^ = 0.568); (**F**) PDXs were differently responsive to cisplatin treatment (5 mg/Kg once a week for three weeks), models were considered responsive when at least a partial response (PR, median of four tumors) was reached. Mean Overall Survival of patients treated after surgery and from whom a responsive PDX was derived (R-treated) were higher than that of patients from whom a non responsive PDX was derived (NR-treated; 27.667 ± 7.753 months n = 3 and 8.5 ± 3.019 months n = 6, respectively). MCR: maintained complete response; CR: complete response; PR: partial response; SD: stable disease; PD: progression of disease.

Pathology examination and molecular analyses of the established PDX models and their parental tumours were carried out to confirm the accuracy of these *in vivo* models. Immunohistochemistry analysis of 27 PDXs confirmed the parental tumour diagnosis corresponding to 20 ADC, 4 SCLC and 3 SCC. Interestingly, the same profile was maintained for more than ten passages in mice (Fig. 1A, Table 3). In the 20 ADC, comparative analysis of histologic patterns, stroma, necrosis percentage and immunohistochemistry markers (TTF-1, p40, vimentin, Ki-67 antigen and synaptophysin) showed a general maintenance of the relevant profiles over several passages in mice. In particular, heterogeneity of histologic patterns in parental tumour was maintained in the corresponding LcPDXs, although there was in late passages (P > 10) a slight tendency for solid pattern to prevail and for acinar and cribriform patterns to be under-represented. Indeed, solid pattern was appreciable in 6 primary tumours, in 7 PDXs at P ≤ 10 and in 8 PDXs at P > 10; acinar pattern in 4, 3 and 2 and cribriform pattern in 6, 5 and 4, respectively (Table 3 and Supplementary Figure 3). Stromal component was generally lower in LcPDXs compared to parental tumours, but a higher level of stromal cells percentage was present in both early (P ≤ 10) and late stage (P > 10) PDXs than in cell line-derived XG (Fig. 1C, Table 3 and Supplementary Figure 3).

**Table 3:** Patients and tumours histopathological analysis.

	All PDX		PDX p > 10*		
	Human n = 27	Mouse (p < 10) n = 27	Human n = 10	Mouse (p < 10) n = 10	Mouse (p > 10) n = 10
***Diagnosis(1)***					
ADC	20	20	9	9	9
SCLC	4	4	1	1	1
SCC	3	3	0	0	0
	**All ADC**		**PDX > 10 (ADC)**		
	**Human n = 20**	**Mouse (p < 10) n = 20**	**Human n = 9**	**Mouse (p < 10) n = 9**	**Mouse (p > 10) n = 9**
***Pattern (1)***					
SOLID	12	11	6	7	8
TRABECOLAR	1	0	NA	NA	NA
LEPIDIC	3	0	3	0	0
SPINDLE CELLS	2	4	1	1**	1**
ACINAR	10	7	4	3	2
CRIBRIFORM	11	7	6	5	4
MUCINOUS	1	2	1	2	2
PAPILLAR	8	2	4	1	1
MICROPAPILLAR	5	4	3	2	2
COLLOID (GELATINOUS)	1	1	NA	NA	NA
SIGNET CELLS	0	1	0	1	1
***Vessels (1)***					
GLOMERULOID	3	4	0	1	1
THIN	4	3	3	1	1
NO	20	20	7	8	8
***Average stromal percentage***	24 ± 12.39	14 ± 8.95	22 ± 12	15 ± 7	10 ± 7
***Average necrosis percentage***	21 ± 20	15 ± 13	21 ± 20	11 ± 9	23 ± 16
***Markers (1)***					
SYNAPTOPHYSIN	4	1	1	1	0
TTF-1	16	9	6	4	4
P40	2	2	2	2	2
VIMENTIN	7	12	4	6	8
KI67	20	20	9	9	9

(1) Number of positive models; *Analysis of 10 PDXs that reached P > 10 in mouse; **Resulting from a pattern loss in one PDX and a pattern acquisition in another PDX.

Analysis of marker expression highlighted that, apart from a general similarity (Fig. 1B), there was a slight decrease of TTF-1 and an increase in vimentin in PDX derived from ADC. Indeed, TTF-1 was expressed in 6 primary tumours, 4 LcPDX at P ≤ 10 and 4 LcPDX at P > 10;and vimentin in 4 primary tumours, 6 LcPDX at P ≤ 10 and 8 LcPDX at P > 10. Moreover, for LT63, LT220 and LT268 complete loss of TTF1 and marked acquisition of vimentin and Ki67 labelling index was observed in mice (Fig. 1D, Table 3 and Supplementary Figure 3). In addition, an analysis of specific tumour cellular subpopulations showed that CD133^+^ cancer initiating cells (CICs) were maintained in LcPDXs with a content similar to that observed in primary tumours (Fig. 1E).

#### Genetic profile

**Figure 2:**
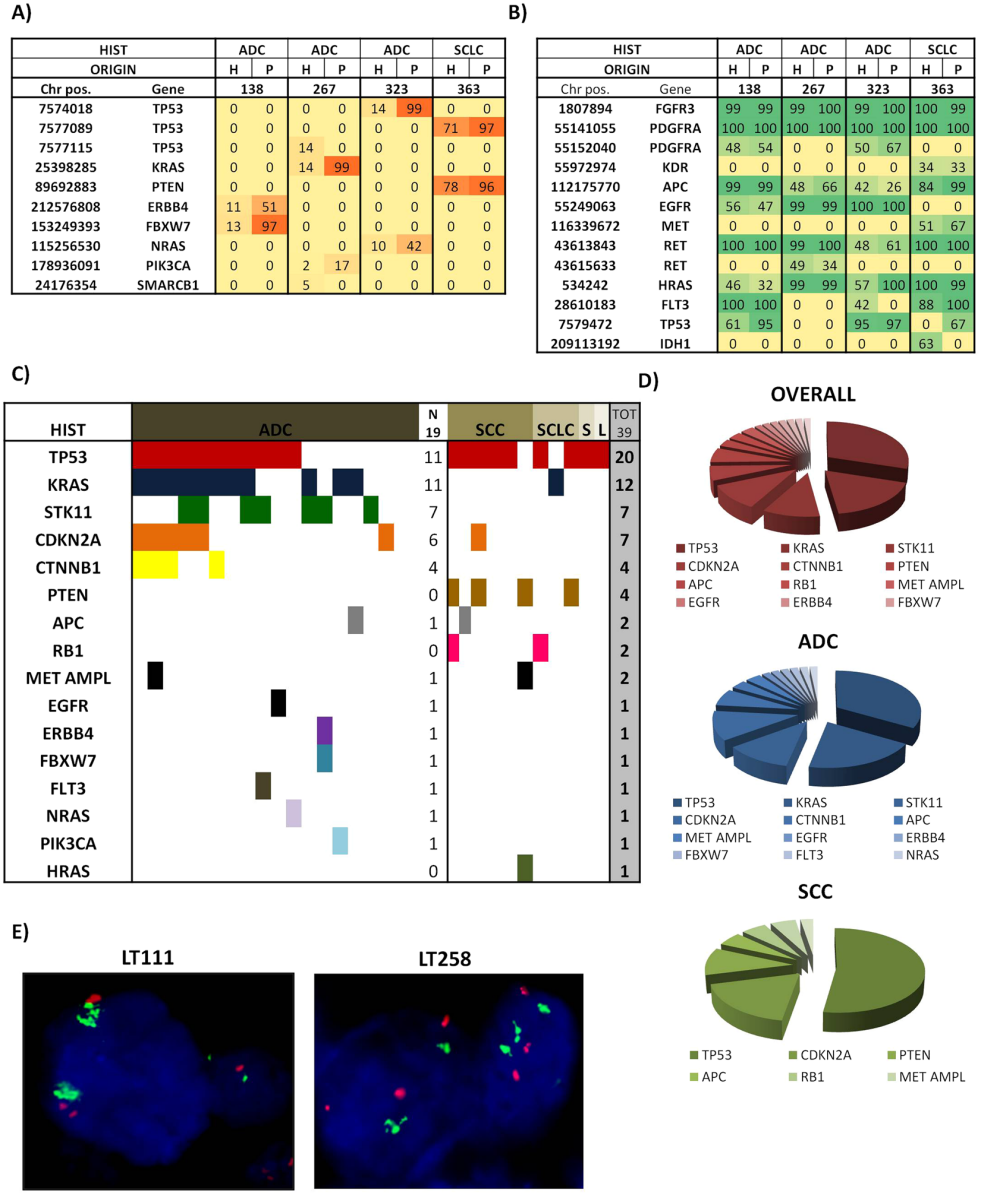
PDX genetic profile. (**A**) Comparison of human tumour DNA and PDXs DNA showed that non synonymous alterations found in parental tumours were generally found also in PDXs but with a higher allelic frequency, whereas synonymous alterations were maintained without increase in allelic frequency (**B**). (**C**,**D**) Table and pie graph show frequency of mutations identified in PDXs platform. (**E**) FISH analysis of two MET amplified PDXs. ADC = Adenocarcinoma, SCC = Squamous Cell Carcinoma.

To further prove the identity of LcPDXs with primary tumours, we performed targeted mutation sequencing using a panel of 50 genes frequently mutated in human cancers and FISH analysis for MET gene amplification. NGS analysis was carried out on 30 different models (19 ADC, 6 SCC, 2 SCLC, 1 sarcomatoid and 1 large cell carcinoma). Overall, *TP53* mutations were identified in 20 PDXs (67%); *KRAS* in 12 PDXs (40%); *STK11* and *CDKN2A* in 7 PDXs (23%); *CTNNB1* and *PTEN* in 4 PDXs (13%); *APC*, *RB1* and *MET* amplification in 2 PDXs (7%); *EGFR*, *ERBB4*, *FBXW7*, *FLT3*, *NRAS*, *PIK3CA* and *HRAS* in 1 PDX (3%). Interestingly, *KRAS*, *STK11*, *CTNNB*1, *EGFR*, *ERBB4*, *FBXW7*, *FLT3*, *NRAS* and *PIK3CA* mutations were exclusively found in ADC, whereas *HRAS*, *PTEN* and *RB1* were exclusively found in the other histotypes (Fig. 2). No mutations were found in two ADC PDXs (LT220 and LT268) (Supplementary Figure 4). Moreover, DNA of 4 LcPDXs was compared to the correspondent human tumours. All mutations identified in PDXs were confirmed in parental tumours, with the exception of LT267 (human tumour mutated in *TP53*, *KRAS*, *PIK3CA* and *SMARCB1*, whereas PDX was mutated in *KRAS* and *PIK3CA* only, Fig. 2A), with a general increase in the frequency of mutated alleles being observed in LcPDXs. Interestingly, also synonymous polymorphisms were maintained in PDX models with an allelic frequency similar to that observed in human tumours (Fig. 2B).

#### Response to treatment

Eighteen PDX models were characterized for their responsiveness to cisplatin. Five of them (responders) reached at least a partial response (PR, median of four tumors) upon treatment, whereas in the other 13 a progression of the disease (PD) was always appreciable (non responders). Interestingly, overall survival (OS) of patients from whom responder PDXs were derived was higher than that of patients which gave rise to non-responders PDXs. (Fig. 1F and Supplementary Tables 4, 5 and 6).

All these observations strengthened the relevance of PDXs as models closely recapitulating original parental tumours.

### PDX grafting is associated with patients survival

**Figure 3:**
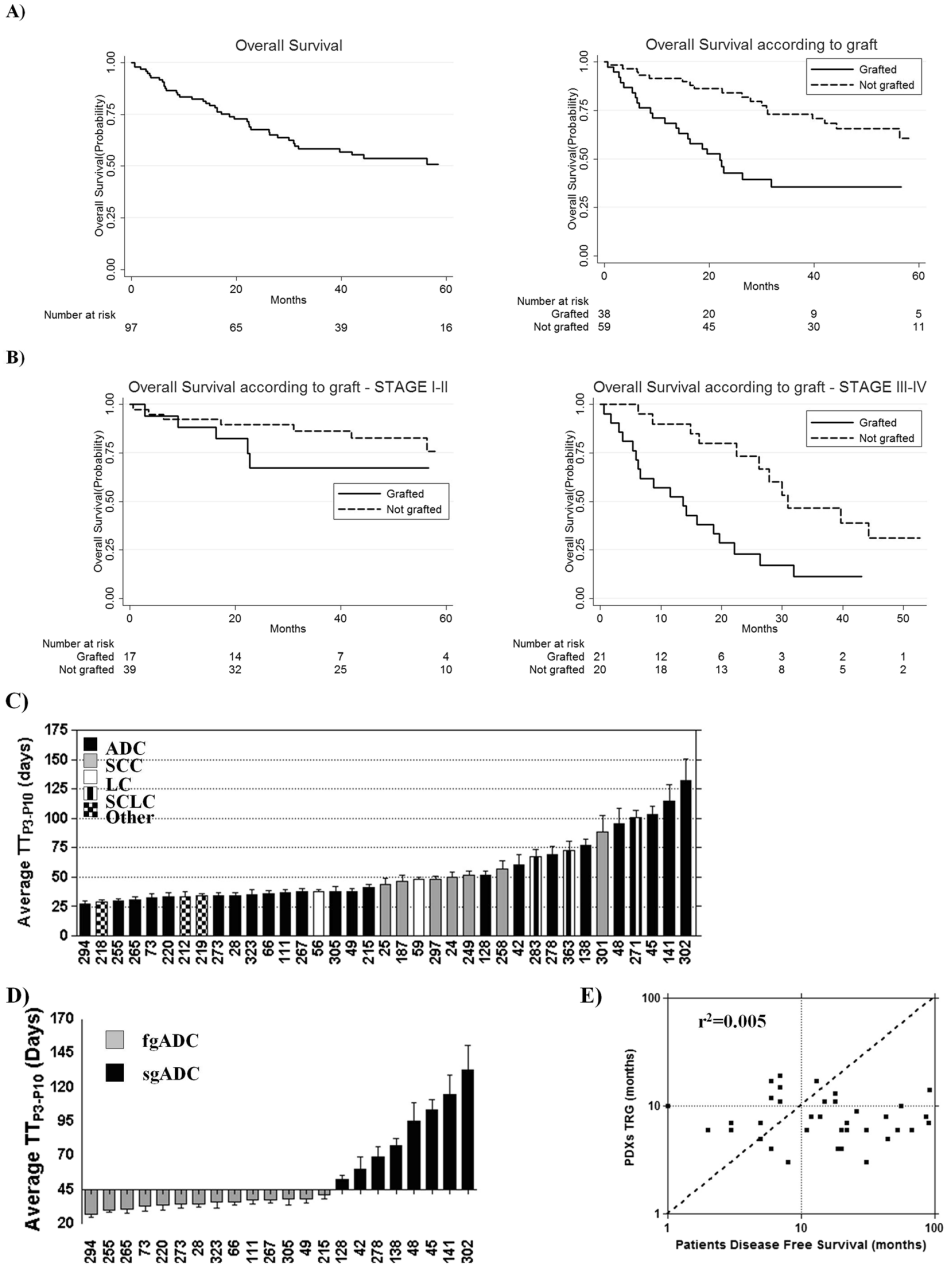
PDX grafting as prognosis determinant and PDXs growth characteristics. (**A**) 5-years OS of all patients involved in PDX platform establishment. 5-years OS of patients from whom PDX was successfully established (grafted) was significantly lower than OS of patients whose tumour sample did not give rise to a PDX (not grafted) (Log-rank test p = 0.0008). (**B**) 5-years OS was lower for stage III/IV patients than stage I/II patients (20%; 95% CI, 10–36% and 73%; 95% CI, 56–85%, respectively). Interestingly, stage III/IV patients with grafted PDX still maintained a significantly lower OS than not grafted (Log-rank test p = 0.0027) whereas stage I/II patients with grafted PDXs showed only a slight tendency towards a lower than not grafted 5-years OS. (**C**) Average time to transplantation (from P3 to P10, TT_P3–P7_) for each PDX. ADC = Adenocarcinoma, SCC = Squamous Cell Carcinoma. (**D**) ADC could be divided in two distinct groups based on their growth characteristics, fgADC = fast growing ADC with TT < 44.95 days and sgADC = slow growing adenocarcinomas with TT > 44.95 days (44.95 days corresponded to the average GT of all PDXs). (**E**) Patient’s DFS and PDXs grafting time (GT, time a model needs to stabilize in mouse and reach P3) were not linearly correlated (n = 38, p = 0.678, r^2^ = 0.005), and for 23 PDXs DFS was higher than GT.

Clinical characteristics of patients were analyzed to investigate PDXs take determinants. Association of sex, age, smoking habits, lung functionality parameters (FEV1, FEV1/FVC and COPD) and clinical outcome with tumour grafting capability were examined. As shown in Table 1 and Table 2, a worse outcome was significantly associated with an increased likelihood of grafting (p = 0.006). An increased grafting probability was observed also for higher stage tumours (p = 0.041 for Stage II, III, IV vs. Stage I and p = 0.038 for Stage III,IV vs. Stage I,II) and tumours with higher CD133^+^/CXCR4^+^/EpCAM^−^ subpopulation (p = 0.019) whereas a trend towards increased grafting for tumours with SUV_max_ higher than 8 (p = 0.084) was observed (Table 1). The correlation between clinical outcome and PDX grafting was further analyzed using Kaplan Meier and Cox analyses. At the time of the present analysis, 42 patients died and the OS rate at 5 years was 51% (95% CI, 39–62%). Interestingly, OS at 5 years was significantly worse (p = 0.0008) in patients with grafted PDXs (36%; 95% CI, 20–52%) than those with not grafted PDXs (61%; 95% CI, 43–74%) (Fig. 3A), also considering tumour stage (73%; 95% CI, 56–85% in stages I/II vs 20%; 95% CI, 10–36% in stages III/IV, p < 0.0001) (Fig. 3B). In multivariable Cox analysis, grafting PDXs and tumour stage significantly influenced OS (Table 2), with HR for death being 2.59 (95% CI, 1.36–4.94) for grafted *versus* not grafted LcPDXs, and 5.59 (95% CI, 2.74–11.40) for stages III/IV *vs*. stages I/II. Age, sex and SUV did not affect OS. Similar results emerged when considering disease-free survival (Table 2).

These data highlight that the capability of tumour samples to establish LcPDXs is an inherent characteristic of primary tumours linked to the biological aggressiveness potential, thus acting as an adverse prognostic marker.

### Grafted PDX may act as “Avatars” for personalized treatment

We further analyzed LcPDX growth characteristics, categorizing our platform based on average time to transplantation (TT- time from implant to explant) in the first 10 passages (Fig. 3C). Average TT for all LcPDX was 44.95 days; average TT for SCC-derived LcPDXs felt almost all around this value, SCLC-derived LcPDXs displayed a slower TT, whereas ADC-derived LcPDXs were split into two distinct groups (faster growing, fgADC and slower growing, sgADC, Fig. 3D). Although no tumour or patient characteristics clearly discriminated fgADC from sgADC, sgADCs were enriched in Stage I tumours (37.5% vs. 4.4% in sgADC and fgADC respectively) and in tumours bearing CTNNB1 mutations (33.3% vs. 14.3% in sgADC and fgADC respectively) and were mainly derived from heavy smokers (Supplementary Tables 2 and 3).

In order to clarify if a co-clinical approach could have been feasible for LcPDXs, we analyzed their growth characteristics according to patient survival, and found no correlation between the time required to reach P3 (Time to Reach Grafting - TRG) and the patient DFS (Fig. 3E), indicating that faster LcPDXs were not necessarily derived from patients with lower OS or DFS. Indeed, for 24/38 (63.2%) LcPDXs (9 Stage I, 6 Stage II and 9 Stage III/IV), TRG was less than DFS of the relevant patients (Fig. 3E). Thus, these LcPDX may have been exploited for meaningful drug testing, before tumour recurrence occurred in patients. Interestingly, this “Avatar” approach could have been put to use to give insights for the treatment of 25.5% (13/51) patients with progression of disease: 16.7% (1/6) Stage I, 33.3% (4/12) Stage II and 24.2% (8/33) Stage III/IV (Table 4).

**Table 4:** PDXs as “Avatars” (DFS > TRG) in relation to patients follow-up.

Stage	PD/D	DFS > TRG	NED	DFS > TRG	Total	DFS > TRG
***Overall***						
I	6	1 (16,7)	30	8 (26,7)	36	9 (25)
II	12	4 (33,3)	9	2 (22,2)	21	6 (28,5)
III	33	8 (24.2)	7	1 (14,3)	40	9 (22,5)
***Grafted PDXs***						
I	1	1 (100)	8	8 (100)	9	9 (100)
II	6	4 (66.7)	2	2 (100)	8	6 (75)
III	19	8 (42.1)	1	1 (100)	20	9 (45)

PD: Progression of Disease; D: Death; NED: No Evidence of Disease; DFS: Disease Free Survival; TRG: Time to reach grafting.

## Discussion

We reported here the establishment (with 39.2% grafting rate) of a large platform of lung cancer PDXs. Although the study population represents less than 10% of all patients who underwent mediastinal biopsy or pulmonary resection with curative intent for primary lung cancer during the same period, there was no selection by major prognostic factors, and median survival of the 97 patients providing PDXs samples was very similar to the one of the other 953 patients (47 vs 57 months). These models recapitulated all the features of original tumours. In particular, tumour architecture was maintained in our PDXs, with stromal content and main histological patterns being similar to parental tumours at least in early passages (P ≤ 10). Interestingly, cellular patterns linked to non aggressive lung cancer subtypes (i.e lepidic)^25^ were lost in our models, whereas a slight tendency toward a selection for more solid, vimentin-expressing tumours was observed in late mouse passages. This suggests at the one side that grafted models could be preferentially derived from more aggressive tumours and at the other side that a tumour evolution towards a higher aggressiveness was appreciable also during PDX passages in mouse. A tumour evolution was strongly suggested by LT63, LT220 and LT268 behaviour in mouse, indeed in these models an epithelial to mesenchimal transition (EMT) was appreciable, with loss of TTF-1, acquisition of vimentin and Ki67 expression. Interestingly, LT220 and LT268 were the only two analyzed tumours for which no mutations were detected, suggesting either that they could carry other rare mutations or that they are not cell-autonomous and their tumour growth and progression are mainly dependent on tumour adaptation to microenvironment (ME). These data suggest a progressive adaptation of PDXs to murine ME. Since PDXs fully recapitulated primary tumour characteristics, it can be argued that these models could also recapitulate the natural history of tumours. Indeed, in our models, the cross-talk between tumour (human) and microenvironmental (murine) cells seemed to produce a selective pressure that advantaged solid patterns, vimentin expression and eventually complete EMT. Thus, although PDXs did not retain original tumour ME and therefore are not suitable for investigating non-cell autonomous (stromal and immune) drivers of tumour evolution^26^, our observations suggest that murine ME may be partly able to vicariate human tumour ME also in driving malignant progression. Thus, LcPDXs may be an operational model to investigate stromal mechanisms underlying cancer evolution towards dissemination (EMT) and aggressiveness and can therefore be also suitable for preclinical studies involving ME (stroma and innate immunity)-directed treatments.

The analysis of clinico-pathological characteristics indicated a good correlation between PDX establishment and survival (OS and DFS). A multivariable Cox analysis confirmed an higher HR for grafted PDXs also when adjusting for Stage, SUV_max_, Sex and Age. These data confirmed that aggressive tumours have an advantage in terms of grafting capability, as suggested by pathology analysis and as already reported for early stage lung cancer PDXs^19^. A trend for metabolically more active tumours (SUV_max_ > 8) to graft in mice was also observed. A low primary tumour SUV_max_ was reported as a predictor of long-term survival and as a tool to identify NSCLC patients without lymph node involvement^27,28^. Lung tumour mortality is mainly due to metastasis development, and it has been reported that PDXs derived from lung cancer brain metastasis showed a higher take rate compared to PDXs derived from primary tumours^23^. However, we found no correlations between PDX establishment and TNM staging of primary tumour. On the contrary, we found a tendency towards a higher grafting capability for tumours according to the presence of CD133^+^/CXCR4^+^/EpCAM^−^ cells. We previously reported the heterogeneity of CD133^+^ cancer initiating cells^29^ and in particular the capability of the CD133^+^/CXCR4^+^/EpCAM^−^ subpopulation to sustain tumour dissemination^30^. All these data suggest that aggressiveness of grafted tumours may mirror an inherent biological trait of tumours and that grafting capability may depend on the presence of cells that are able to seed in a non orthotopic soil. Interestingly, LcPDXs showed enrichment in mutation rate of *TP53* in all tumour types and of *KRAS* in ADC where the frequency of mutations was almost double than that expected from literature data (57.9% *KRAS* mutated PDX vs 25–35% *KRAS* mutated ADC^31^) whereas *EGFR* mutations were rarely present. The assumption of *KRAS* mutations as prognostic markers in lung ADC is controversial^32–36^, but the high rate of *KRAS* mutations in grafted PDXs seem to support this hypothesis.

Grafted PDXs were categorized based on growth rate and ADC-derived PDX were divided in two groups (fgADC and sgADC). No patient or tumour characteristics could discriminate these two groups, probably because of the low number of models (14 fast and 8 slow) under evaluation. Additional work will clarify if these two groups were derived from tumours or patients with distinct biological traits, in particular to confirm a prevalence of stage I tumours from heavy smokers or *CTNNB1*-mutated tumours to give rise to sgADC or a slightly higher tendency of *KRAS*-mutated tumours to give rise to fgADC. As a role of aberrant WNT signalling in response to cigarette smoke has already been reported^37,38^, sgADCs could represent tumours of heavy smokers with impaired WNT/beta catenin pathway. Accordingly, sgADCs might represent a tumour subgroup with decreased tumour-microenvironment cross-talk capability, either because of the lack of smoke-induced lung inflammation in mouse or due to a particular mutational status.

One of the main advantages of establishing LcPDXs is that parental tumour traits can be conserved over time, making biological studies available even after patient death (Supplementary Figure 4). This represents a unique opportunity to investigate specific therapies in tumours with different pathological and molecular features as already demonstrated, utilizing our NSCLC PDX platform, for bevacizumab-treated LKB1 mutated tumours^39^. However, to exploit these models as being “avatars” for personalized patient therapy, the time required to stabilize them in mouse has necessarily to be less than DFS and, due to clinical aggressiveness of lung cancer, the use of PDXs for developing personalized therapy strategies is generally unfeasible. This issue coupled with the generally low grafting rate, allows mainly to exploit the broadest spectrum of different mutations of LcPDXs to investigate the efficacy of targeted therapies rather than to implement co-clinical approaches. Nonetheless, we here first show that 63.2% of grafted models was stabilized in mice before occurring of tumour recurrences, thereby offering a chance to personalized treatment in approximately one fourth of all lung cancer patients involved in this study and, interestingly, in one third of Stage II (and one fourth of Stage III) patients with progression of disease.

In conclusion, we reported the establishment of a wide panel of LcPDX, which accurately mirrored primary tumours in terms of subtype, growth pattern, genotype, metabolic activity and cancer stem cell composition. Moreover, PDX take rate correlated with patient OS and DFS, tumour stage, SUV, and presence of CD133^+^/CXCR4^+^/EpCAM^−^ cells, consistent with more aggressive tumours prone to disseminate. Our LcPDX platform, beside recapitulating a broad spectrum of lung cancer-related mutations useful to test targeted therapies, could also be suitable for developing personalized co-clinical studies and investigating tumour-microenvironment cross-talk. All these observations strengthen the relevance of LcPDXs as an operational and robust pre and co-clinical model closely mirroring parental tumours, which could play a strong clinical role in biological and pharmacologic studies.

## Material and Methods

### Patients Selection

Tumour samples were collected from May 2006 to July 2013, with a sterile procedure in the operating room, after mediastinal biopsy (9 patients) or anatomical pulmonary resection, such as pneumonectomy, lobectomy or segmentectomy (88 patients). These patients represent 7% (9/135) of all mediastinal biopsies, and 10% (88/915) of all anatomical resections for primary lung cancer, performed at the “Fondazione IRCCS Istituto Nazionale dei Tumori” during the same period. Samples of primary NSCLC were obtained from patients undergoing surgical resection, who gave their informed consent after approval from the Internal Review and the Ethics Boards of the Fondazione IRCCS Istituto Nazionale Tumori and all methods were performed in accordance with istitutional guidelines and regulation.

### PDXs establishment

PDXs were established as described in ref.^5^. PDXs models were propagated for three rounds in mice (P1–P3) before to be considered stabilized and then frozen in a solution of 90% FBS and 10% DMSO and stored in liquid nitrogen. The experimental protocol was approved by the C.E.S.A. (Ethical Committee for Animal Experimentation, of the National Cancer Institure Foundation), and animal experimentation was performed following guidelines drawn-up by C.E.S.A. according to ref.^40^.

### *In vivo* treatments

Experiments were carried out in groups of 4 mice, bearing a PDX in each flank. Mice were treated once a week for three weeks with 5 mg/Kg Cisplatin (Teva) or vehicle. Tumour growth was followed by calliper once a week and results were analyzed using GraphPad Prism software. Response Rate was calculated as follows (adapted from PPPTP testing and analysis methods: https://ctep.cancer.gov/content/docs/PPPTP_TESTING_AND_ANALYSIS_METHODS.pdf): maintained complete response (MCR, score: 10): maximal growth inhibition vs controls >50% and tumor volume (Tv) < 100 mm^3^ at the end of treatments; complete response (CR, score: 8): MGI > 50% and Tv < 100 mm^3^ at least in 1 time point; partial response (PR, score: 6): MGI > 50% and Tv always > 100 mm^3^; stable disease (SD, score:4): MGI < 50% and increase in initial volume (iiV) <25%; progression of disease 2 (PD2, score:2): MGI < 50% and iiv >25% and tumor growth delay (TGD) > 1.5; PD1 (score: 0): MGI < 50% and iiv > 25% and TGD < 1.5. TGD was calculated as (time to event)/(average time to event of controls), assuming an event as the time a tumor needs to triple its starting volume.

### NGS analysis

DNA was quantified with NanoDrop 2000 (Thermo Fisher Scientific) and aliquots of 100 ng were shipped to Genewiz laboratories (http://www.genewiz.com) for targeted NGS analysis using the Ion AmpliSeq™ Cancer Hotspot Panel v2 (Thermo Fisher Scientific). Raw sequence data were trimmed, classified with Xenome software^41^ to eliminate as many mouse reads as possible, aligned in NextGENe and checked for variants using NextGENe v 2.3.4.4 and v2.4.1 software (Softgenetics).

### FISH Analysis

Fluorescence *in situ* hybridization analysis was performed in 24 μ-thick paraffin sections by counting at least 100 tumour cells. Briefly, to assess MET amplification a commercially available probe ZytoLight® SPEC MET/CEN 7 Dual Colour Probe (localized at chromosome 7q31 locus) was utilized according to manufacturer’s instructions. MET gene amplification was defined as the ratio of MET/Chromosome 7 equal or greater than 2.0, or the presence of clusters in at least 10% of analyzed tumour cells.

### Immunohistochemistry

Thyroid transcription factor-1 (TTF1), as a marker for lung adenocarcinoma^42^; synaptophysin for neuroendocrine differentiation^43^; DNp63/p40 (henceforth simply p40) for squamous cell carcinoma^44^; vimentin for epithelial-mesenchymal transition in lung cancer^45^ and Ki-67 for cell proliferation activity^46^ immunohistochemistry was performed on both LcPDXs and paired surgical specimens, which had been formalin fixed and paraffin embedded according to standard histopathology methods. Briefly, three-four micron-thick sections were made react with the relevant primary antibodies for 30 min (TTF1, clone 8G7G3/1, Dako, Glostrup, Denmark, dilution 1:2000 with EDTA buffer unmasking at pH 8 for 30 min; synaptophysin, clone DAK-SYNAP, Dako, dilution 1:200 with EDTA buffer unmasking at pH 8 for 15 min; p40, polyclonal, Calbiochem Millipore, San Diego, CA, USA, dilution 1:3000 with EDTA buffer unmasking at pH 8 for 40 min; vimentin, clone V9, Dako, dilution 1:400 with citrate buffer unmasking at pH6 for 15 min; Ki-67 antigen, clone MIB-1, Dako, dilution 1:400 with EDTA buffer unmasking at pH 8 for 15 min). Sections were then incubated with a commercially available detection kit (EnVision™ FLEX+, Dako) in an automated immunostainer (Dako Autostainer System, Dako). The specificity of all reactions was double-checked replacing the primary antibody with a non-related mouse immunoglobulin at a comparable dilution or using normal serum alone. Positive and negative controls were adopted as required. Results were rendered semi-quantitatively as the percentage of labelled cells showing convincing cytoplasm (synaptophysin, vimentin) or nuclear (TTF1, p40, Ki-67) decoration on the basis of the specific gene product being investigated.

### Flow Cytometry

Single-cell suspensions (10^6^ cells) were incubated in staining solution containing 1% BSA, 2 mM EDTA and phycoerythrin (PE)-conjugated anti human CD133/1 (Miltenyi Biotech) and/or Allophycocyanin (APC)-conjugated anti human CD184 (BD Pharmingen™) and/or fluorescein isothiocyanate (FITC)- conjugated anti EPCAM (Miltenyi Biotech). To assess PDXs stromal content, single-cell suspensions (10^6^ cells) were washed and incubated in staining solution containing 1% BSA, 2 mM EDTA and AlexaFluor®488-conjugated anti human HLA (BD Pharmingen™) and PreCP-eFluor 710- conjugated anti murine MHC(H-2Kd) (eBioscience). Samples were acquired by FACS Calibur and analyzed with FlowJo_V10 software.

### Statistical analysis

Continuous variables were presented as mean values ± standard deviation (SD) and median with inter-quartile range (IQR), and categorical variables as numbers and percentages. Comparisons among groups for continuous variables were performed using a two-sided Student’s t-test for normally distributed variables and a two-sided Wilcoxon’s rank-sum test for variables not conforming to a normal distribution, and for categorical variables using contingency table analysis with the Chi-square test. The primary end-points of the study were overall survival (OS) and disease free survival (DFS). For each end-point, the time to event occurrence was computed from the date of surgery to the date when the event was recorded, or was censored at the date of last follow-up assessment in event-free patients. Hazard ratios (HR) of OS and DFS and the corresponding 95% confidence intervals (CIs) according to age, sex, tumour stage, SUV, and grafting of tumour were estimated using Cox proportional hazard models. A multivariable analyses was performed including terms for all these factors in the same Cox model. Survival curves were estimated using the Kaplan–Meier method and were compared by the log-rank test. Graphical evaluation by Schoenfeld residual plots indicated that the model assumptions concerning proportional hazards were appropriate. All tests were two-sided and a p-value of less than 0.05 was taken as statistically significant. Statistical analyses were performed using SAS 9.2 (SAS Institute, Cary, NC) and the figures were obtained using STATA 11.0 (StataCorp LP, College Station, TX) statistical software.

## Electronic supplementary material

Supplementary Information
