# Author Correction: Establishment of patient derived xenografts as functional testing of lung cancer aggressiveness

**DOI:** 10.1038/s41598-018-21279-z

**Published:** 2018-02-27

**Authors:** Massimo Moro, Giulia Bertolini, Roberto Caserini, Cristina Borzi, Mattia Boeri, Alessandra Fabbri, Giorgia Leone, Patrizia Gasparini, Carlotta Galeone, Giuseppe Pelosi, Luca Roz, Gabriella Sozzi, Ugo Pastorino

**Affiliations:** 1 Tumor Genomics Unit, Department of Experimental Oncology and Molecular Medicine, Fondazione IRCCS Istituto Nazionale dei Tumori, Milan, Italy; 2 Department of Pathology and Laboratory Medicine, Fondazione IRCCS Istituto Nazionale dei Tumori, Milan, Italy; 3 Thoracic Surgery Unit, Department of Surgery, Fondazione IRCCS Istituto Nazionale dei Tumori, Milan, Italy; 4 Department of Clinical Sciences and Community Health, Università degli Studi di Milano, Milan, Italy

Correction to: *Scientific Reports* 10.1038/s41598-017-06912-7, published online 27 July 2017

The original version of this Article contained errors in the spelling of the authors Massimo Moro, Giulia Bertolini, Roberto Caserini, Cristina Borzi, Mattia Boeri, Alessandra Fabbri, Giorgia Leone, Patrizia Gasparini, Carlotta Galeone, Giuseppe Pelosi, Luca Roz, Gabriella Sozzi & Ugo Pastorino which were incorrectly given as Moro Massimo, Bertolini Giulia, Caserini Roberto, Borzi Cristina, Boeri Mattia, Fabbri Alessandra, Leone Giorgia, Gasparini Patrizia, Galeone Carlotta, Pelosi Giuseppe, Roz Luca, Sozzi Gabriella & Pastorino Ugo respectively.

These errors have now been corrected in the PDF and HTML versions of the Article, and in the accompanying Supplementary Information file.

