# Supplementary material for: Establishment of patient derived xenografts as functional testing of lung cancer aggressiveness
Source: Sci Rep. 2017 Jul 27;7:6689. doi: 10.1038/s41598-017-06912-7 (PMC5532258; doi:10.1038/s41598-017-06912-7)
Supplement: Supplementary file 1 — Supplementary Information [file 41598_2017_6912_MOESM1_ESM.pdf]

Establishment of patient derived xenografts as functional testing of lung cancer aggressiveness

Massimo Moro[1], Giulia Bertolini[1], Roberto Caserini[1], Cristina Borzi[1], Mattia Boeri[1], Alessandra Fabbri[2], Giorgia Leone[2], Patrizia Gasparini[1], Carlotta Galeone[5], Giuseppe Pelosi[2], Luca Roz[1], Gabriella Sozzi[1#*] and Ugo Pastorino[3#]*

Supplementary Informations

Supplementary Table 1: PDXs samples Freeze/Thawing success percentage

PDX	P	N. thawed	N.grafted	Take (PDX)	Take (samples)
**48**	15	4	4	Y	100%
**48**	16	4	0	***N***	0%
**66**	3	4	4	Y	100%
**66**	51	4	4	Y	100%
**66**	52	2	2	Y	100%
**73**	4	4	4	Y	100%
**111**	12	4	4	Y	100%
**111**	13	4	4	Y	100%
**111**	13	6	6	Y	100%
**111**	28	4	4	Y	100%
**128**	8	4	4	Y	100%
**128**	9	4	1	Y	25%
**138**	11	4	0	***N***	0%
**187**	4	4	2	Y	50%
**215**	4	4	4	Y	100%
**215**	13	4	2	Y	50%
**215**	17	6	0	***N***	0%
**220**	11	4	2	Y	50%
**305**	4	4	4	Y	100%
**363**	6	4	4	Y	100%
**TOTAL**		**82**	**59**	**85.0%**	**72.0%**

Supplementary Table 2. fg PDX vs sgPDX mutations (ADC)

		Stage	TP53	KRAS	STK11	CDKN2A	CTNNB1	EGFR	FLT3	NRAS	PIK3CA	APC
SLOW GROWING	128	IIIA	■	■	■	■						
	42	IB	N/A	N/A	N/A	N/A	N/A	N/A	N/A	N/A	N/A	N/A
	278	IIIA			■							
	138	IA										
	48	IB	■	■		■	■					
	45	IIIA	N/A	N/A	N/A	N/A	N/A	N/A	N/A	N/A	N/A	N/A
	141	IIIA	■	■			■					
	302	IIIA	■					■				
	TOT		4	3	2	2	2	1	0	0	0	0
	%		66,7	50	33,33	33,333	33,333	16,7	0	0	0	0
		Stage	TP53	KRAS	STK11	CDKN2A	CTNNB1	EGFR	FLT3	NRAS	PIK3CA	APC
FAST GROWING	294	IIA				■						
	265	IV	■		■				■			
	73	IIA		■	■							
	220	IIIA										
	273	IIIB	■	■	■							
	28	IIA	N/A	N/A	N/A	N/A	N/A	N/A	N/A	N/A	N/A	N/A
	323	IIA	■							■		
	66	IIIA	■	■	■	■						
	255	IA	■	■		■	■					
	111	IIB	■	■		■	■					
	267	IIIA		■							■	
	305	IIIA		■								■
	49	IIIA	N/A	N/A	N/A	N/A	N/A	N/A	N/A	N/A	N/A	N/A
	215	IV	■	■								
	TOT		7	8	4	4	2	0	1	1	1	1
	%		58.3	66,7	33.3	33.3	14.3	0	7,1	7,1	7,1	7,1
ALL ADC	TOT		11	11	6	6	4	1	1	1	1	1
	%		61.1	61.1	33,3	33.3	22.2	5,6	5,6	5,6	5,6	5,6

missing growth data for 1 ADC

Supplementary Table 3. Patients' and tumors' characteristics, according to PDX's growing characteristics (ADC).

	PDX's Growth		
	**fgADC (n=14)*** **	**sgADC (n=8)*** **	**p-value***
Patients characteristics			
Sex			
Female	8(57.1)	5 (62.5)	*0.584*[1]
Male	6 (42.9)	3 (37.5)	
Age			
Mean (sd)	63.3 (11.3)	64.9 (9.4)	*0.945*[2]
Smoking habits			
Never	3 (21.4)	1 (12.5)	
Ex	5 (35.7)	3 (37.5)	*0.799*[1]
Current	6 (42.9)	4 (50.0)	
Pack years			
≤40 pack-years	9 (64.3)	5 (62.5)	0.642[1]
>40 pack-years	5 (35.7)	3 (37.5)	
Mean (sd)	32.2 (19.4)	37.8 (18.8)	**0.031**[2]
FEV1**			
Mean (sd)	96.8 (21.0)	95.1 (14.1)	0.938[2]
FEV1/FVC**			
Mean (sd)	71.3 (13.2)	73.7 (9.2)	0.508[3]
Chronic obstructive pulmonary disease			
No	5 (38.5)	5 (71.4)	0.349[1]
Yes	8 (61.5)	2 (18.6)	
Outcome			
Mortality			
Alive	6 (42.9)	3 (37.5)	1.000[1]
Dead	8 (57.1)	5 (62.5)	
Disease			
No	4 (28.6)	3 (37.5)	0.510[1]
Yes	10 (71.4)	5 (62.5)	
Missing	0	1	
Tumor characteristics			
Standardized Uptake Value (SUV)			
≤5	2 (16.7)	2 (25.0)	0.535[1]
>5	10 (83.3)	6 (75.0)	
≤8	3 (25.0)	4 (50.0)	0.251[1]
>8	9 (75.0)	4 (50.0)	
Missing	2	0	
Mean (sd)	10.2 (5.6)	9.95 (6.3)	0.313[2]
Stage			
I	1 (4.4)	3 (37.5)	
II	5 (38.5)	0 (0.0)	*0.060*[1]
III / IV	8 (57.1)	5 (62.5)	
TNM Staging**			
N=0	3 (25.0)	4 (57.1)	0.182[1]
N>0	9 (75.0)	3 (42.9)	
Missing	2	1	
CD133⁺ cells[4]			
> =1%	6 (46.2)	4 (57.1)	0.182[1]
< 1%	7 (53.8)	3 (42.9)	
Missing	1	1	
$CD133^+/CXCR4^+/EpCAM^-$ cells[4]			
Yes	7 (70.0)	3 (60.0)	0.566[1]
No	3 (30.0)	2 (40.0)	
Missing	4	3	

*P for between group comparison: [1]Fisher's Exact test; [2]Wilcoxon's rank-sum test; [3] Student's t-test; [4] PDX's values; **2 missing values.***missing growth data for 1 ADC
